# Carbapenems drive the collateral resistance to ceftaroline in cystic fibrosis patients with MRSA

**DOI:** 10.1038/s42003-020-01313-5

**Published:** 2020-10-22

**Authors:** Maria Celeste Varela, Melanie Roch, Agustina Taglialegna, Scott W. Long, Matthew Ojeda Saavedra, Warren E. Rose, James J. Davis, Lucas R. Hoffman, Rafael E. Hernandez, Roberto R. Rosato, Adriana E. Rosato

**Affiliations:** 1 Department of Pathology and Genomic Medicine, Center for Molecular and Translational Human Infectious Diseases Research, Houston Methodist Research Institute, Houston, TX USA; 2 School of Pharmacy, University of Wisconsin-Madison, Madison, WI USA; 3 Argonne National Laboratory (DOE), Lemont, IL USA; 4 Computation Institute, University of Chicago, Chicago, IL USA; 5 Department of Pediatrics and Department of Microbiology, University of Washington, Seattle, WA USA; 6 Center for Clinical and Translational Research, Seattle Children’s Research Institute, Seattle, WA USA; 7 Houston Methodist Cancer Center, Houston Methodist Hospital, Houston, USA; 8 Present Address: Riverside University Health System-Medical Center, 26520 Cactus Avenue, Moreno Valley, CA 92555 USA

**Keywords:** Microbiology, Preclinical research

## Abstract

Chronic airways infection with methicillin-resistant *Staphylococcus aureus* (MRSA) is associated with worse respiratory disease cystic fibrosis (CF) patients. Ceftaroline is a cephalosporin that inhibits the penicillin-binding protein (PBP2a) uniquely produced by MRSA. We analyzed 335 *S. aureus* isolates from CF sputum samples collected at three US centers between 2015–2018. Molecular relationships demonstrated that high-level resistance of preceding isolates to carbapenems were associated with subsequent isolation of ceftaroline resistant CF MRSA. In vitro evolution experiments showed that pre-exposure of CF MRSA to meropenem with further selection with ceftaroline implied mutations in *mecA* and additional mutations in *pbp1* and *pbp2*, targets of carbapenems; no effects were achieved by other β-lactams. An in vivo pneumonia mouse model showed the potential therapeutic efficacy of ceftaroline/meropenem combination against ceftaroline-resistant CF MRSA infections. Thus, the present findings highlight risk factors and potential therapeutic strategies offering an opportunity to both prevent and address antibiotic resistance in this patient population.

## Abstract (web summary)

Varela, Roch et al. find that resistance of preceding isolates to carbapenems is associated with the subsequent isolation of methicillin-resistant *Staphylococcus aureus* (MRSA) resistant to ceftaroline. Addition of meropenem reduces the cystic fibrosis MRSA’s resistance to ceftaroline in a pneumonia mouse model, suggesting a strategy to control antibiotics resistance.

## Introduction

Cystic fibrosis (CF) is a life-shortening, autosomal recessive genetic disease that primarily affects Caucasians^1^. CF affects many organs, most notably the lungs where a thick mucus is produced in the airways and predisposes to pulmonary colonization and infection by a number of pathogens. According to the CF Foundation Patient Registry, *Staphylococcus aureus* is currently the most common bacteria identified in CF lungs in the US, having surpassed *Pseudomonas aeruginosa* in prevalence in the early 2000s^2,3^. CF patients frequently receive antibiotics to treat these infections, which often results in the selection of antibiotic-resistant organisms, including methicillin-resistant *S*. aureus (MRSA). In 2018, more than 70% of CF patients in the United States had at least one positive culture for *S. aureus* including over 25% of MRSA^3–5^. Emerging research has demonstrated that MRSA infections are associated with substantial morbidity (decline lung function) and mortality among individuals with underlying chronic diseases such as CF, where metabolic adaptations may facilitate the establishment of persistent infections^6–9^.

The changing epidemiology of CF infections and varying resistance to commonly used antibiotics, and in particular the involvement of MRSA are influencing the choice and the efficacy of anti-infective treatments. In this contex, ceftaroline (CPT), a fifth-generation cephalosporin with potent bactericidal activity against MRSA, is considered as a clinically relevant therapeutic option^10^. Among the β-lactam antibiotics, CPT has the particular ability to inhibit both the native PBPs and the acquired PBP2a, the principal determinant of high-level β-lactam resistance in MRSA by allosteric opening of its active site^11^. CPT has been approved by the Food and Drug Administration (FDA) for the treatment of community-acquired bacterial pneumonia (CABP)^12^. Furthermore, CPT demonstrated a high clinical cure rate (>70%) in pneumonia caused by both MSSA and MRSA^13^. CPT is active against MRSA with reduced susceptibility to vancomycin (VAN), linezolid (LZD), and daptomycin (DAP)^14,15^. The Clinical and Laboratory Standards Institute (CLSI) has established CPT breakpoints of ≤1 mg/L for susceptible MRSA strains, 2 mg/L for intermediate strains, and ≥4 mg/L for resistant strains^16,17^. Unfortunately, resistance to this newly licensed drug has already been described including in CF strains^18,19^. We previously reported three isogenic CF MRSA isolates including one highly resistant to CPT (CPT-HR) (MIC > 32 mg/L) that were isolated between 2012 and 2013^19,20^. In another CF center, one CPT intermediate resistance (CPT-IR) was reported (MIC 4 mg/L) in a patient who received repeated treatment by different β-lactam including ceftaroline^18^ Interestingly, retrospectives studies conducted in Spain, Greece, Switzerland, and Thailand, found CPT-IR strains isolated prior to the launch of CPT in the market, and they, therefore, were not selected by direct CPT exposure^21–23^. Their resistant phenotype was associated with mutations in the *mecA* gene, which encode PBP2A, in the allosteric non-penicillin-binding domain (nPBD)^23–25^. Results from a study analyzing 14,902 clinical isolates sampled in the USA from 2008 to 2010 found that surrogate β-lactam markers can predict CPT activity against *S. aureus*^26^. Carbapenems, imipenem (IPM) or meropenem (MER), MICs ≤8 mg/L were shown to be the best predictor of CPT susceptibility^26^.

Based on Jones et al.^26^ and our own observations, we hypothesized that pre-exposure to carbapenems, notably meropenem, which is used for the treatment of *P. aeruginosa* CF infections, may favor the selection of CPT-R strains upon subsequent CPT exposure. If true, pre-exposure to carbapenems would be a key risk factor for CPT resistance in CF MRSA. In this study, we tested 335 CF *S. aureus* isolates collected from diverse geographical CF centers in the US. We characterized them phenotypically and determined their genotypic relationships, identifying risk factors for CPT-R CF MRSA detection. We also performed in vivo analysis of potential therapeutic options for the treatment of CPT-R CF MRSA infections. In this study, to the best of our knowledge, we provide for the first time mechanistic evidence linking collateral resistance between pre-exposure of MRSA-infected CF patients to MER and the development of CPT resistant in these isolates. In addition, our findings describe promising approaches to both predict and prevent CPT treatment failure in CF patients with MRSA respiratory infection.

## Results

### Carbapenems-pre-exposure favors CPT resistance

To assess the phenotypic relationship between meropenem and CPT, a collection of 335 CF *S. aureus* isolates that included both wild-type (WT) and small colony variants (SCVs) was assembled from retrospective archives at three academic medical institutions with large CF populations: The University of Washington CF Center (Seattle, WA), UW Health (Madison, WI), and Houston Methodist Hospital (Houston, TX). These isolates were cultured from CF respiratory samples between 2015 and 2018, comprising 162 non-duplicated CF MRSA and 173 CF MSSA. We defined the susceptibilities of these isolates to CPT and MER by both E-test and microdilution MIC. Although there are no CLSI interpretive criteria for meropenem with *S. aureus*, we conservatively categorized strains by meropenem MIC and defined them as MER-susceptible (MER-S) equating to cefoxitin breakpoint of ≤4 mg/l. In addition, this categorization allowed us to use carbapenems as surrogate of ceftaroline activity as previously recommended by Jones et al.^26^.

Based on these criteria, we found that 34.2% (*n* = 56) of the MRSA isolates were susceptible to both MER and CPT (MICs 0.25–1 mg/L), while the remaining 65.8% were MER-resistant (MER-R; MIC 2–32 mg/L). Importantly, of the MER-R group, 23% were also resistant to CPT (MIC 4 to >32 mg/L) (Table 1). Therefore, 35% of the CF MRSA isolates in this study were concomitantly resistant to MER and CPT. Considering MER is generally intended to target *P. aeruginosa* when used to treat CF patients, the association between MER and CPT resistance in *S. aureus* observed here indicates that MER may play a role in selecting CPT resistance primarily in *P. aeruginosa* co-infected patients.

**Table 1:** Percent of CF clinical MRSA isolates from three US CF centers that were resistant to meropenem (MER)- and ceftaroline (CPT).

CF *S. aureus* clinical isolates (*n* = 335; MSSA = 173; MRSA = 162)		
	Meropenem (MER)	Ceftaroline (CPT)
MRSA (*n* = 162)	MER-S: 34.2% (*n* = 56)	CPT-S: 34.2% (*n* = 55)
		CPT-IR: 0%
		CPT-HR: 0%
	MER-R: 65.8% (*n* = 106)	CPT-S: 42.85% (*n* = 69)
		CPT-IR-HR: 23% (*n* = 37)

MER-susceptibility was defined by equating to cefoxitin breakpoint of ≤4 mg/L.

*S* susceptible, *IR* intermediate resistance, *HR* highly resistant.

### Phylogenetic relatedness of MER/CPT-resistant isolates

**Fig. 1:**
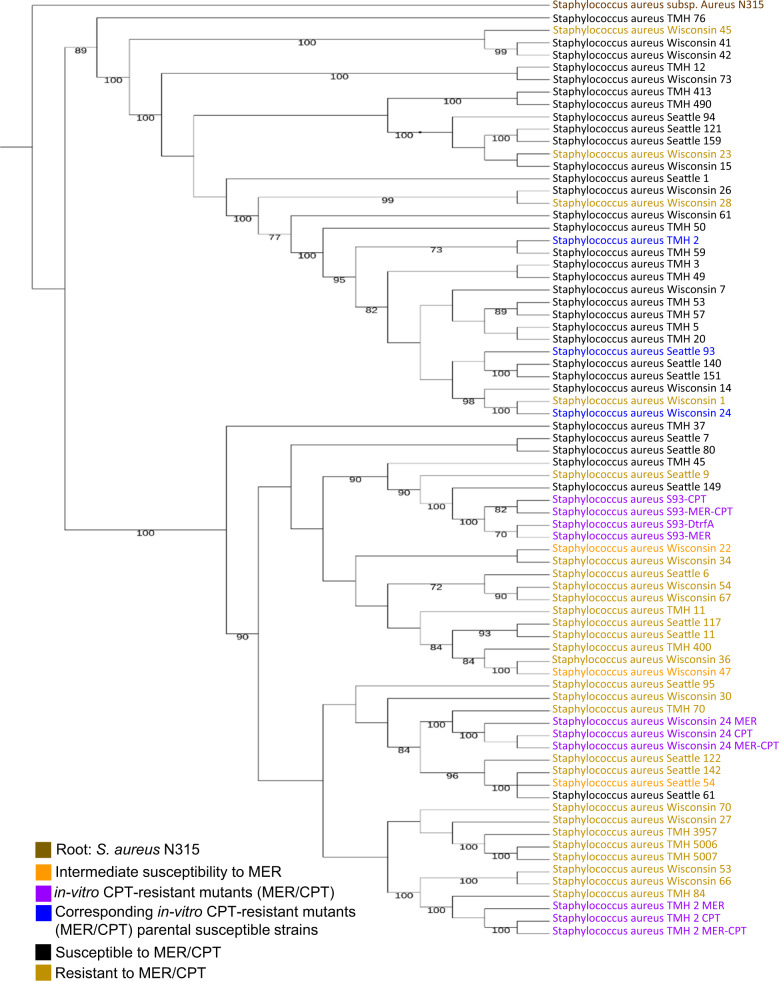
Phylogenetic tree generated with genome sequence alignments corresponding to 75 of the 162 clinical MRSA cystic fibrosis isolates from three CF centers in the USA, including representative in vitro generated MER pre-exposed CPT-resistant mutants SA 93, WIS 24, and TMH 2. Root: *S. aureus* N315. Isolates resistant to both meropenem and ceftaroline are highlighted in green; in vitro ceftaroline-resistant mutants (MER/CPT) are highlighted in purple and its corresponding susceptible parents in blue, isolates with intermediate susceptibilities to meropenem are highlighted in orange, and those susceptible to meropenem and ceftaroline are highlighted in black.

To determine whether MER-resistant CF MRSA isolates were either phylogenetically related and predictors of CPT resistance, phylogenetic tree of *S. aureus* CF genomes was built using the “Codon Tree” pipeline in the PATRIC phylogenetic tree service^27^. To this end, genome sequence alignments corresponding to 75 of the 162 clinical MRSA cystic fibrosis isolates from three CF centers in the USA, including representative in vitro generated MER pre-exposed CPT-resistant mutants SA 93, WIS 24, and TMH 2, were used. This analysis showed that all CF isolates sequenced here divided into two distinct clusters distinguished those isolates resistant expressing high resistance to MER (MIC ≥ 32 mg/L) from those susceptible to this drug. Consistent with our hypothesis that MER exposure may be a risk factor for CPT resistance, most MER-R isolates were also resistant to CPT (MIC 1.5–2 mg/L); as shown in Fig. 1, both were located in the bottom clade, together with the prototype strain TMH5007. In contrast, the CF MRSA that were susceptible to both agents clustered together in the upper clade. These results also suggest that CF MRSA that are MER- and CPT-resistant do not belong to a specific clonal type, arguing against transmission or a common source. Although ST-5 and ST-8 seem to be the most prevalent clonal types found in our collection of CF MRSA, additional clonal types such as ST75, ST42, ST-105, ST-474, ST-512, and ST1065 were also represented.

To explore the emergence of CPT resistance among MRSA lineages exhibiting high-level resistance to MER, we examined whether certain specific non-synonymous mutations (SNPs) in MER-resistant CF MRSA isolates tended to occur prior to the emergence of CPT resistance. To perform this analysis, we aligned the bacterial genomes of isolates exhibiting MER MIC ≥ 32 mg/L and CPT MIC ≥ 2 mg/L (i.e., TMH70, WIS 27, WIS 53, SEA 142, Fig. 1) against the *S. aureus* N315 control strain. All the analyzed isolates exhibiting high-level MER resistance, MIC ≥ 32 mg/L were located in the same lower clade of the phylogenetic tree have common genetic mutations (Table 2). Two of the non-synonymous mutations were found in the PBP2 gene, resulting in amino-acid changes Cys197Tyr and Ser707Leu. PBP2, which is also required for the full expression of methicillin resistance, is a high-molecular weight class A PBP with both transpeptidase and transglycosylase domains^28^. In the presence of high concentrations of methicillin, PBP2 is completely acylated, though its β-lactam-insensitive transglycosylase domain is still required for resistance, implying that PBP2a and PBP2 functionally cooperate during growth in the presence of β-lactams^29^. Additional nonessential proteins such as SgtB were found to be mutated at position Met88Val. SgtB is involved in peptidoglycan synthesis, such as the monofunctional glycosyltransferases SgtA and SgtB^30^. Of interest, SgtB mutations have been reported to increase resistance to β-lactams^31^.

**Table 2:** Mutations identified commonly in highly MER-R (32 mg/L; CPT 2 mg/m = L) CF clinical isolates of MRSA belonging to the lower clade of the phylogenetic tree shown in Fig. 1.

Accesion #	Gene	Function	Amino-acid change
SA0991	*mutS2*	Recombination inhibitory protein MutS2	Val252Ile
SA1283	*pbp2*	Penicillin-binding protein PBP2, staphylococcal type	Cys197Tyr
SA1283	*pbp2*	Penicillin-binding protein PBP2, staphylococcal type	Ser707Leu
SA1691	*sgtB*	Monofunctional biosynthetic peptidoglycan transglycosylase	Met88Val
SA1964	*fmtB*	FemD, factor involved in methicillin resistance	Thr641Ile
SA1458	*lytH*	LytH protein involved in methicillin resistance	Gly74Ser
SA0793	*dltA*	D-alanine-poly(phosphoribitol) D-alanylation of teichoic acids	Gly253Val
SA0796	*dltB*	D-alanyl transfer protein DltB	Ile264Lys
SA0107	*spa*	Protein A, von Willebrand factor binding protein Spa	Asn234Thr
SA1638	*lukE*	Cytolytic pore-forming protein S component	Lys4fs
SA1097	*hslU*	ATP-dependent hsl protease ATP-binding subunit HslU	Leu93Val
SA1736	*aldH*	Aldehyde dehydrogenase	Glu427Val
SA0912	*qoxB*	Cytochrome aa3-600 menaquinol oxidase subunit I	Glu445Asp

*fs* frameshift.

Accessory genes with known roles in β-lactam resistance were also mutated, notably *fmtB* at position corresponding to Thr641Ile. FmtB is linked to β-lactam resistance and peptidoglycan metabolism, and its inactivation impacts the function of glucosamine mutase GlmM on its substrates *N*-acetylglucosamine or glucosamine^32^. We also found mutations in genes associated with virulence: Lys4fs (frameshift) in *lukE*, which subverts host defenses during *S. aureus* infections; a Asn234Thr mutation in *spa*, which is a target for host inflammation and plays a role in the pathogenesis of staphylococcal diseases^33^; a Glu427Val mutation in *aldH*, which is a metabolics gene that encodes aldehyde dehydrogenase; and a Glu445Asp mutation in *qoxB*, which is a terminal oxidase with roles in electron transport and energy metabolism. Taking these data together, we found that PBP2 mutations were particularly common in all the isolates in this clade, including the MER-R (MIC ≥ 32 mg/L) and CPT-R (MIC 2 mg/L) strains TMH70, WIS 53, WIS 27, SEA 142. In addition, these isolates have mutations in genes associated with virulence, host response, and metabolism.

### MER pre-exposure increases the likelihood of CPT resistance

**Fig. 2:**
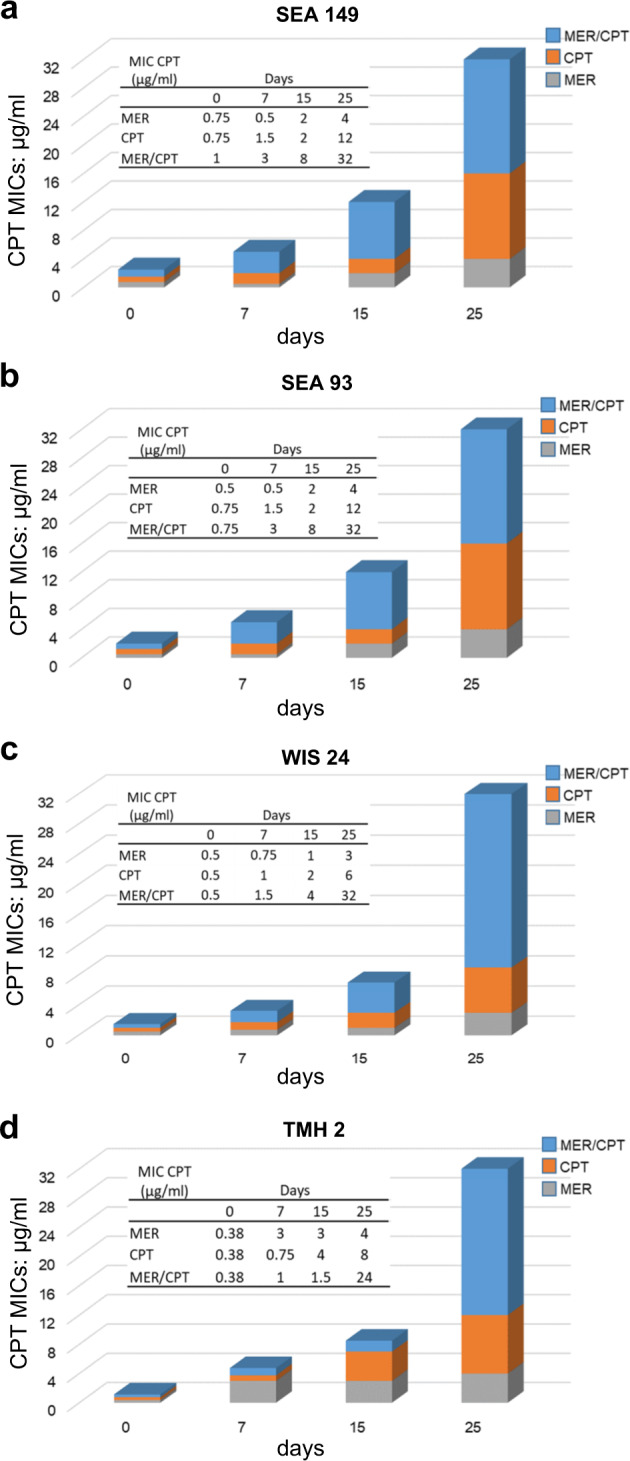
In vitro susceptibilities of MRSA strains from serial passage of CF isolates in ceftaroline. This in vitro evolution experiment was performed for several CF MRSA isolates, SEA 149, SEA-93, TMH 2, and WIS 24 phenotypically susceptible to both; meropenem and ceftaroline from the upper clade of the phylogenetic tree in Fig. 1. The indicated CF MRSA isolates were exposed to either meropenem (MER) or ceftaroline (CPT) alone, or pre-exposed to MER (10 mg/L), in microplates with two-fold increasing sub-inhibitory concentrations of MER or CPT (0.25; 0.5; 1; 2; 4; 8; 16; 32; 64; 128 mg/L). After 24 h of incubation, samples corresponding to the highest antibiotic concentration were used to inoculate new series of plates containing antibiotic dilutions; this daily passage approach was repeated for up to 25 days. Ceftaroline MICs were defined in the resulting mutants at days 0, 7, 15, and 25.

Based on our genotypic findings that PBP2 was primarily mutated in MER-R and CPT-R CF (MIC: 2 mg/L) clinical isolates, and that *pbp1* was mutated in the clinical TMH5007 prototype high level of CPT resistant (CPT MIC 32 mg/L), and that both PBP2 and PBP1 (specific) are targets of carbapenems^34–36^, we hypothesized that pre-exposure to the carbapenems (e.g., MER) currently used in the treatment of *P. aeruginosa* infection in CF patients, favors the selection of subsequent CPT resistance in *S. aureus*. To determine precisely whether pre-exposure to carbapenems is associated to genotypic changes of *pbp2* and *pbp1* in *S. aureus* during CPT acquisition in CF MRSA, we conducted an in vitro evolution experiment by generating CPT-R mutants in a representative number of CF-MRSA strains located in the upper clade of phylogenetic tree that had MER susceptibilities between 0.75 and 1.5 mg/L (MER-S). We passaged MER-S isolates SEA-93, SEA 149, Wis-24, and TMH 2 in progressively higher concentrations of either MER or CPT, from sub- to supra-inhibitory concentrations in both cases, for 25 consecutive days. Under these experimental conditions and when the isolates were directly exposed to MER or CPT alone, we observed an increase in CPT MICs ranging from 0.5 to 12 mg/L (Fig. 2). In contrast, when strains were pre-exposed to MER (10 mg/L) and then progressively exposed to CPT (MER/CPT) for 25 days, an increase of CPT resistance relative to no MER pre-exposure was observed as demonstrated by CPT MICs ≥32 mg/L. Interestingly, WGS and phylogenetic analysis of the mutant recovered strains showed them located at the bottom clade (Fig. 1; purple color strains) together with prototype TMH5007 isolate while their parent strains (MER-S/CPT-S) were located at the upper clade (Fig. 1; blue color strains). These results imply that CPT-HR is favored by pre-exposure to MER (MER/CPT) followed by long-term exposure to CPT.

**Fig. 3:**
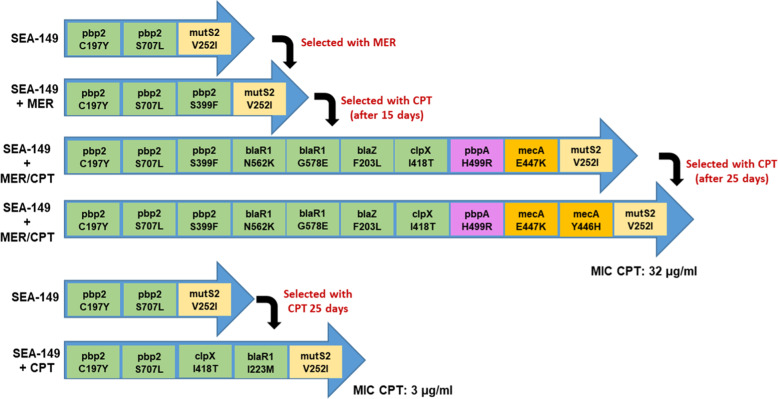
Description of point mutations during the in vitro acquisition of CPT-R through MER pre-exposure in order of their appearance during the exposure of CF MRSA (SEA 149) for 25 days to increasing concentrations of ceftaroline. SA149 + MER/CPT isolate, pre-exposed to meropenem, and subsequently passage to ceftaroline was the only condition that introduces amino-acid changes Y446H in the *mecA* gene.

Experimental evolution of differential mutations in the resulting strains at day 25 were further investigated by comparing the strains that were isolated after passaging of SA149—SA149 CPT, SA149 MER, and SA149 MER/CPT—with the SA149 parent strain. We found that the *mecA* mutation at position Y446H only emerged following exposure to CPT of the pre-exposed MER isolate (identified as SA149 MER/CPT) and was accompanied by an increase in CPT MIC of 32 mg/L. By contrast, no mutation in *mecA* was seen in the SA149 MER or SA149 CPT mutant strains (Fig. 3). Interestingly, in SA149 MER/CPT, we identified non-synonymous mutations in *pbp1* (H499R), as mentioned PBP1 is a selective target of imipenem (carbapenem) in addition to its major role in *S. aureus* cell division and separation^37,38^; additional mutations where found *blaR1* (G578E; N562K) and in *blaZ* (F203L) genes implicated in *mecA* regulation^39,40^. Interestingly, exposure to MER alone (i.e., SA149 MER) was followed by a third mutation into the *pbp2* gene (S399F), while exposure to CPT (i.e., SA149 CPT) was associated with a *clpX* mutation (I418T) (Fig. 3). These data strongly reinforce the notion that pre-exposure of CF MRSA to MER with further selection with CPT implies mutational changes in *pbp1 pbp2*, primary targets of carbapenems in addition to the primary target of CPT, *mecA* which contribute to the emergence of high levels of CPT resistance in CF-MRSA isolates.

### PIP/TAZ and CEF pre-exposure does not select CPT resistance

**Fig. 4:**
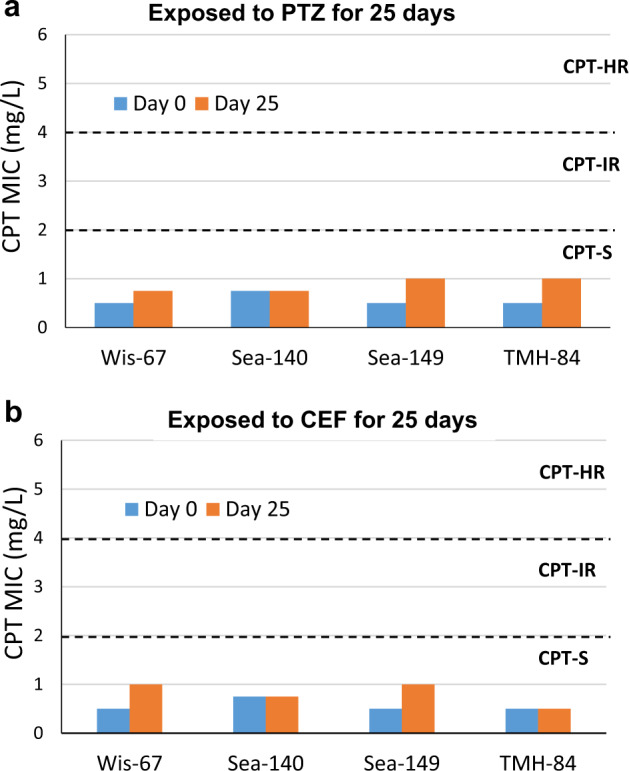
Pre-exposure to PIP/TAZ and CEF does not select CPT resistance. Ceftaroline MIC (mg/L) of four cystic fibrosis MRSA clinical isolates; WIS 67, SEA 140, SEA 149, TMH 84 exposed for 25 days to increasing concentrations of **a** piperacillin-tazobactam (PTZ) and **b** cefepime (CEF).

We assessed whether the observed increase in CPT resistance was specific to MER or shared with other antimicrobials used to treat *P. aeruginosa* infections, specifically piperacillin/tazobactam (PIP/TAZ) and cefepime (CEF). CF MRSA isolates were treated/pre-exposed to PIP/TAZ and CEF by daily passages of cells with increasing concentrations of CPT for 25 consecutive days, as previously described with MER. Pre-exposure of WIS 67, SEA 140, SEA 149, and TMH 84 to PIP/TAZ and CEF resulted in increased MICs (32–128 mg/L) to these antibiotics. The strains were then passaged in increasing concentrations of CPT for 25 days. In contrast to the same approach using MER, we did not observe changes in CPT between days 0 and 25, with values remaining in the susceptible range MICs: 0.5–1 mg/L (Fig. 4).

Together, these observations confirmed that exposure of CF MRSA isolates that were initially MER-susceptible to MER could increase selection for CPT resistance. Importantly, other agents such as PIP/TAZ and CEF, which are also used to treat *P. aeruginosa* infections in CF patients, were not associated with the development of CPT resistance in CF MRSA.

### MER/CPT combination therapy overcomes CPT resistance

**Fig. 5:**
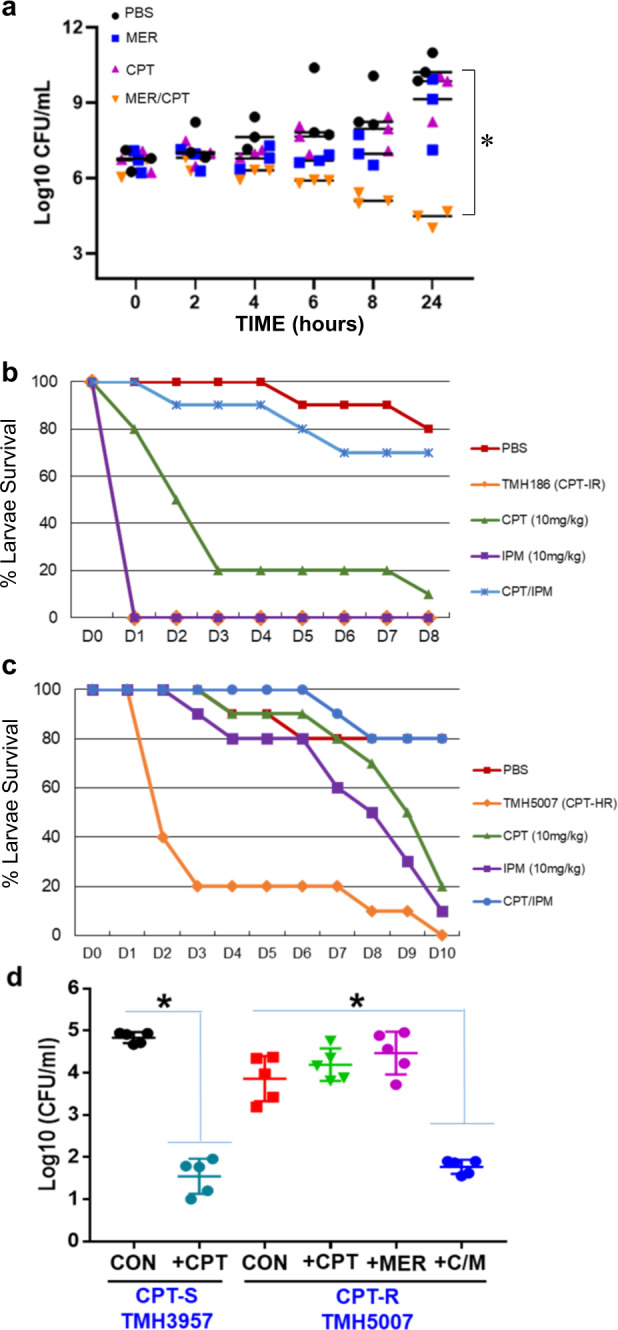
In vitro and in vivo assesment of CPT/MER combination activity against CPT-R MRSA. **a** Time-kill curve of CF MRSA (TMH 5007) with meropenem, ceftaroline, and the combination of ceftaroline and meropenem determined using human peak free serum dose CPT and MER concentrations (16 mg/L); the results represent three independent experiments significantly different, *p* < 0.001. **b**, **c** In vivo infection analysis following treatment of *G. mellonella* larvae inoculated with 3 × 10^6^ of CPT-R (TMH5007). Antimicrobial treatment of MER 10 mg/kg, CPT 10 mg/kg, and combination of MER/CPT was initiated after 2 h of inoculation, as subsequently at 24 and 48 h later. Survival data was plotted using the Kaplan–Meier method. **d** Analysis of ceftaroline efficacy in a mouse pneumonia infection model. Groups of five mice were intranasally inoculated with 1.7 × 10^8^ CFU of either CPT-S (TMH 3957) or CPT-R (TMH5007); after 2 h, mice were treated with intraperitoneal CPT (10 mg/kg), MER (25 mg/kg), or the combination of both, until euthanasia at 48 h. Lungs were collected for quantitation of recovered CFU. Results were expressed in log_10_ of CFU/g of organ; significantly different; *P* < 0.001. CON, control; C/M, treatment combination of CPT/MER.

The inefficacy of monotherapy with either CPT and MER against CF MRSA prompted us to explore potential drug combinations that may prove effective against CPT-R isolates, using CPT-R TMH5007 type strains for testing. We hypothesized that, because MER and CPT do not affect the same targets, concurrent treatment with MER and CPT would be more effective than either drug alone, as we have previously shown with β-lactam combinations against MRSA^41^. First we tested the antimicrobial effectiveness of CPT with MER by in vitro time-kill curves. The analysis was performed in subcultures at 0, 2, 4, 6, 8, and 24 h with an initial inoculum of 1 × 10^6^ CFU/mL, and using CPT and MER at their corresponding human serum concentration with standard IV dosing, 16 mg/L. We found that neither CPT nor MER were individually active against the CPT-HR TMH5007 strain, as demonstrated by recurrent growth at 4–8 h up to 24 h (Fig. 5a). However, CPT in combination with MER was highly effective and resulted in a decrease of ≥3 log_10_ CFU.

As in previous studies by the authors^20,41^ and others^42^, we used the larvae of the greater wax moth (*Galleria mellonella*) as a model host for assessment of the efficacy of antistaphylococcal agents. Groups of larvae (10/group) were inoculated with a bacterial suspension containing either CPT-IR TMH 186 (Fig. 5a) or CP-HR TMH 5007 isolates (Fig. 5b) and incubated for 2 h at 37 °C, after which CPT (10 mg/kg), MER (10 mg/kg), or the combination CPT/MER was administered and the larvae reincubated for 24 h at 37 °C. An uninfected control group received PBS treatment to control for multiple injections. After the first 24 h of incubation, treatment was repeated. Worms were checked daily, and any deaths were recorded for up to 8–10 days.

While groups of both strain-injected worms untreated (only injected with PBS) or treated with either single drug displayed very low survival rates (≤20%), treatment with the CPT/IPM combination resulted in survival rates of between 70 and 80% at days 8–10, TMH186 and TMH 5007, respectively. Uninfected worms treated with PBS showed 80% survival at days 8 and 10, respectively, showing that, while worms injected with TMH5007 displayed very low survival rates when treated with CPT, MER, or vehicle PBS (0–20%), administration of CPT/IPM resulted in drastically increased survival of 70–80% (Fig. 5b, c). The CPT/MER combination was further tested by using a mouse pneumonia model (Fig. 5d). Mice were infected with either the susceptible CPT strain TMH 3957 or the CPT-R TMH 5007 strain. In mice infected with TMH 3957, CPT (10 mg/kg) reduced bacterial density by 3.55 log_10_ CFU (4.95 ± 0.21 to 1.4 ± 0.24), while no changes were observed in TMH5007-infected mice treated with the same CPT dose (4.79 ± 0.19 to 4.33 ± 0.14 (Fig. 5d). Similarly, MER alone did not decrease log_10_ CFU; however, a statistically significant reduction of 4 log_10_ CFU was observed with CPT/MER treatment (4.37 ± 0.22 with MER treatment alone vs. 1.61 ± 0.14 with CPT/MER). These results indicate that the combination of CPT and MER was more effective than either single agent thus representing a powerfully effective treatment for CPT-R MRSA strains. In agreement with data from in vitro time-kill experiments (Fig. 5a), results from both in vivo approaches (Fig. 5b–d) show that the interactions between CPT and MER are synergistic, and may represent a clinically relevant anti-MRSA therapeutic option.

## Discussion

Antibiotic failure and recurrent infections in CF patients are of critical concern. CF patients with chronic *S. aureus* respiratory infections can have frequent respiratory exacerbations that require antibiotic treatments, and it is common for these patients to simultaneously receive multiple antibiotics to treat co-infecting bacteria, including MSSA, MRSA, and *P. aeruginosa*^43^. MER is frequently used to treat *P. aeruginosa* infections^44–46^, and the cephalosporin CPT is increasingly being used against MRSA infections^47–50^. New reports on patients with bacteremia, endocarditis, and MRSA pneumonia treated with CPT have been published and, importantly, most of these MRSA infections failed to respond to previous antimicrobials, highlighting the necessity and utility of CPT^51^.

Our data indicate that the pre-exposure of MRSA-infected CF patients to MER may increase the likelihood of development of CPT-resistant *S. aureus* isolates. Carbapenem MIC values have been shown to be the best predictor of CPT susceptibility^26^. In this study, we retrospectively characterized CF MRSA respiratory isolates from our institution and others around the US in terms of phenotype and genotype. We found that 30% of the CF MRSA isolates we tested were CPT-resistant. Considering that CPT was FDA-approved in 2010^14,15,26,52–54^, i.e. after the collection of these strains, these findings are worrisome. Previous studies indicated that CPT resistance could be associated not just with the source country of specific isolates^19,21,22,24,25^ but also with the MLST profile^55^; conversely, however, the CPT-resistant isolates analyzed in this study had diverse MLST profiles, including ST-8, ST-5 (the most common in the USA), ST-45, ST-72, ST-105, ST-474, ST-512, and ST-1075, indicating no such specificity for sequence type. In addition, isolates in this study tended to be concurrently resistant to both MER and CPT regardless of MLST. More importantly, our study provides evidence that other β-lactam antibiotics that are currently used to treat *P. aeruginosa* infections in CF patients, such as PTZ and CEF^56,57^, do not select for CPT resistance in MRSA, indicating that carbapenems may have a particular tendency among the β-lactams to select for CPT resistance.

We speculate that this unique property of MER may result of its affinity for PBP2 and PBP1. Mutations in this gene may alter PBP2, PBP1 binding affinity of MER^34–36^, which could increase the likelihood of subsequent mutations in *mecA*, given the known importance of the interaction between the transglycosylase domain of PBP2 with PBP2a in conferring resistance to β-lactams^29^ and the known essential role of PBP1 in cell division^37,38^. Using WGS, we showed that CF MRSA isolates that exhibited phenotypes with MER ≥ 32 mg/L and CPT ≥ 2 mg/L clustered by sequence into a specific clade and did not carry non-synonymous SNPs in the *mecA* gene, which encodes PBP2a. However, they did carry non-synonymous mutations in *pbp2* and other genes associated with β-lactam resistance and in maintaining cell wall composition and function (e.g., *fmtB, dtlA, dltB*, and *lytH*). Notably, these mutations were present in all the isolates displaying a MER-resistant (MIC 32 mg/L) and CPT-resistant (MIC 2 mg/L) phenotype. In experimental evolution experiments, we then showed that exposure of these aforementioned isolates to CPT select for strains exhibiting even higher-level resistance to CPT (MIC 32 mg/L), often resulting in mutations in *mecA* at Y466H. These mutations, which accrued sequentially in vitro, were the same as those we identified in the first clinical CF-MRSA strain we reported as CPT-R (TMH5007). It is tempting to speculate that these mutations similarly occurred sequentially in TMH5007.

In support of this hypothesis, in a 2016 clinical case report of a child with CF with both *P. aeruginosa* and MRSA who received 22 courses of CPT over 30 months until clinical failure was declared, the authors concluded that MER was a key factor responsible for increased MRSA CPT MIC over time^18^. Results from another study provide further support: An analysis of 14,902 US clinical MRSA isolates from 2008 to 2010 found that, of several candidate markers tested, MICs ≤8 mg/L to the beta-lactams IPM or MER were shown to be the best predictors of CPT susceptibility^26^. These findings strongly support our hypothesis that MER is a key driver of CPT resistance development in MRSA and, furthermore, provide evidence of the molecular basis associated with this process.

*S. aureus* population structures vary because of adaptation to local environments and antibiotic use^23^; for these reasons, we expanded our isolate collection to include several sites in the US beyond our home center in Houston, Texas. Our findings indicate that the relationship observed here between MER and CPT is independent of geography or MRSA sequence type. We also observed that CPT-R CF clinical isolates tended to carry non-synonymous mutations in genes associated with virulence (e.g., *spa* and *lukE*); accordingly, these isolates tended to have lower pathogenicity in a wax worm model of infection. Similarly, it has been observed that a substantially larger proportion of MRSA isolates from patients with chronic infections, including CF, carry *spa* differential protein repeats than do isolates from patients with acute infections or healthy carriers^33^.

In conclusion, we have identified collateral resistance observed between two classes of antibiotics commonly used to treat CF patients with *P. aeruginosa* and *S. aureus* infections. Our data also provide a likely mechanism for these concurrent resistance patterns. Based on our results, we propose that the combined administration of CPT and MER may be useful in patients carrying multiresistant MRSA. Chronic CF respiratory infections are remarkably persistent and difficult to treat due to a number of factors that impact antimicrobial effectiveness, including increased antimicrobial clearance, requiring higher antimicrobial doses than in many other clinical scenarios. CF patients are often exposed to multiple courses of broad-range antibiotic regimens; our results indicate that these practices may predispose to the development of resistance to CPT, among our most useful antibiotics for treating MRSA. The deeper understanding of the mechanisms leading to CPT resistance we describe here, and the in vitro efficacy of the combination-therapeutic approach we propose, identify promising avenues for preventing and addressing CPT resistance in the vulnerable CF patient population.

## Methods

### Bacterial strains and antimicrobial susceptibility testing

*S. aureus* strains were isolated from sputum samples from cystic fibrosis patients, either WT or small colony variant phenotypes, and were obtained from three academic medical institutions with large CF populations: the Center of Global Infectious Diseases (Seattle, WA), UW Health (Madison, WI), and Houston Methodist Hospital (Houston, TX). Strains were selected from stored isolate banks by investigators at these institutions. These strains were identified from patient sputum at the time of clinical care, characterized in the microbiology laboratory, and stored at −80 °C until analysis. Only one strain per patient was included.

Ceftaroline was provided by Allergan (Dublin, Ireland) and meropenem (MER) was purchased at Ark Pharm (Illinois, USA). Minimal inhibitory concentration (MIC) to CPT, MER, piperacillin-tazobactam (PTZ), cefepime (CEF), and vancomycin (VAN) were determined by E-test (BioMerieux, Marcy l’Etoile, France) and broth dilution and interpreted according to CLSI guidelines^16,17^. For CPT a strain is considered susceptible at ≤1 mg/L, intermediate at 2 mg/L, and resistant at ≥4 mg/L. There are no CLSI interpretive criteria for meropenem with *S. aureus*, however, we conservatively categorized strains by meropenem MIC and defined them as MER-susceptible (MER-S) equating to cefoxitin breakpoint of ≤4 mg/L for purposes of this study. In addition, this categorization allowed us to use carbapenems as surrogate of ceftaroline activity as previously recommended by Jones RN et al.^26^. Additional antimicrobial susceptibility testing was performed for rifampicin (RIF), ciprofloxacin (CIP), chloramphenicol (CM), oxacilin (OXA), cefoxitin (FOX), meropenem (MER), piperacillin-tazobactam (PTZ), and cefepime (CEF) by the Kirby–Bauer method with Sensi-disks (BD, Franklin Lakes, NJ) and interpreted according to the CLSI guidelines^16^. Specifically, susceptibility testing was used to evaluate strain phenotype and to follow-up the strains identity (check for the absence of cross-contamination in microplates) during long-term passages experiments and in addition to WGS analyses^16^. The reference strains ATCC25923 and ATCC29213 was used as a quality control for antibiotic susceptibility testing following CLSI guidelines.

### Ceftaroline in vitro mutant from pre-exposed MER CF-MRSA strains

CPT resistance selection was performed in representative CF-MRSA clinical strains for each of the CF centers involved in this study; TMH 2, SEA-93, SEA 149, and WIS 24 by progressive selection. Briefly, microplates containing two-fold increasing sub-inhibitory concentrations of MER or CPT (0.25; 0.5; 1; 2; 4; 8; 16; 32; 64; 128 mg/L) were inoculated with 1 × 10^5^ CFU/ml of each clinical strain. After 24 h of incubation at 37 °C, 50 µL of samples from the well containing the highest antibiotic concentration and still turbidity were used to inoculate a new series of microplates containing antibiotic dilutions. This progressive daily passage was done for 25 days. Phenotypic analyses to ensure identity of recovered mutants were performed at days 0, 7, 15, and 25. At the end of the experiment, the genotypic identity of the strains was confirmed by PFGE and whole-genome sequencing (WGS). The recovered MER mutants treated with MER 10 mg/L and further selected by CPT progressive exposure were named MER/CPT mutants.

### In vivo effectiveness of the CPT/MER combination

Mice of the NOD.Cg-Prkdcscid Il2rgtm1Wjl/SzJ (NSG) strain were used in this study (The Jackson Laboratory, Bar Harbor, ME). Animals were fed with standard small laboratory animal diet, and no restrictions were applied. Groups of five mice were infected with 50 µL of inoculum administered to the nares and inhaled into the lung. Treatment commenced 2 h after challenge with intravenous (IV) injections of ceftaroline 10 mg/kg, meropenem 25 mg/kg, or both agents every 12 h; these regimes are comparable to human exposure. After 48 h of treatment, the mice were euthanized by carbon dioxide asphyxiation. Untreated control mice were assessed at the time of treatment initiation. Following euthanasia, the lungs were harvested using sterile technique, homogenized, diluted, plated, and incubated, and viable plate counts were recorded. Data were expressed as the mean change in log_10_ CFU/lungs for three mice per treatment group compared to the organism burden at the start of therapy.

### Animal ethics statement

All animal studies were approved by the Institutional Animal Care and Use Committee of the Houston Methodist Research Institute, protocol numbers AUP 0320-0023 and AUP 1018-0061. To ensure protection and proper manipulation of animals, experiments were performed by trained personnel at the animal facility of the Houston Methodist Research Institute.

### Statistical analysis

Three therapy groups were considered for this study with five animals per group: control infected untreated; infected + ceftaroline; infected + meropenem; infected + ceftaroline/meropenem. A total of ~25 mice were required for these experiments, including an extra number for any problems. Statistical significance was determined by unpaired *t*-test for differences in mean tissue titers for the combination therapy group versus the means for each of the single-drug therapy groups. As there are two pairwise comparisons, the *p* value to reject the null hypothesis was 0.025 with a power of 0.80 to detect 2 log_10_ CFU/g difference and a standard deviation of 1.5.

### Genome sequencing and phylogenetic analysis

Genomic DNA of a representative number of CF clinical strains (68 strains) from in vitro selected mutants was extracted from overnight cultures grown in Mueller-Hinton Broth at 37 °C by using the DNeasy Blood & Tissue Kit (Qiagen, Hilden, Germany). Libraries were prepared from purified DNA using Nextera XT DNA Library Preparation Kit (Illumina, San Diego, CA) and sequenced (50 nucleotide reads) with Hiseq 2000 instrument at the Epigenetics and Genomic Laboratory at Weill Cornell Medical College, NY.

Reads for 75 *S. aureus* sequenced genomes were assembled using The Pathosystems Resource Integration Center (PATRIC) genome assembly service^27^ using the “Full Spades” pipeline, which uses Bayes Hammer^58^ for error correction and SPAdes for the assembly^59^. All genomes were annotated using the PATRIC annotation service in May of 2019^60^. A phylogenetic tree was built for all of the genomes using the “Codon Tree” pipeline in the PATRIC phylogenetic tree service^27^. The tree was built by first finding conserved protein families for the set of genomes. Then 100 conserved, singly occurring, proteins were randomly selected and aligned using MUSCLE^61,62^, and trimmed with Gblocks^63^. The corresponding nucleotide sequences were aligned using the BioPython “codon align” function^64^ and the tree was built from concatenated nucleotide and protein alignments using RaxML^65^.

### Reporting summary

Further information on research design is available in the Nature Research Reporting Summary linked to this article.

## Supplementary information

Description of additional supplementary files

Supplementary Data 1

Supplementary Data 2

Supplementary Data 3

Reporting Summary

## Data Availability

The datasets generated during and/or analyzed during the current study are available in the following repositories. All raw genome sequence data have been submitted to the Pathosystems Resource Integration Center (PATRIC) database and are publicly available. DNA sequencing data is available at Sequence Read Archive (SRA; https://www.ncbi.nlm.nih.gov/sra) under bioproject accession PRJNA635117. All the data produced for this study including in vitro generated mutants are available. Source data underlying plots shown in figures are provided in Supplementary Data 1–3.

## References

[1] Paranjape SM, Mogayzel PJ (2014). Cystic fibrosis. Pediatr. Rev..

[2] Mogayzel PJ (2014). Cystic Fibrosis Foundation pulmonary guideline. pharmacologic approaches to prevention and eradication of initial *Pseudomonas aeruginosa* infection. Ann. Am. Thorac. Soc..

[3] Foundation, C. F. *Cystic Fibrosis Foundation Patient Registry. 2018 Annual Data Report*. (Bethesda, Maryland, 2019).

[4] Knapp EA (2016). The cystic fibrosis foundation patient registry. Design and methods of a national observational disease registry. Ann. Am. Thorac. Soc..

[5] Salsgiver EL (2016). Changing epidemiology of the respiratory bacteriology of patients with cystic fibrosis. Chest.

[6] Zemanick ET, Hoffman LR (2016). Cystic fibrosis: microbiology and host response. Pediatr. Clin. North Am..

[7] Besier S (2007). Prevalence and clinical significance of *Staphylococcus aureus* small-colony variants in cystic fibrosis lung disease. J. Clin. Microbiol..

[8] Sanders DB (2010). Failure to recover to baseline pulmonary function after cystic fibrosis pulmonary exacerbation. Am. J. Respir. Crit. Care Med..

[9] Dasenbrook EC (2011). Update on methicillin-resistant *Staphylococcus aureus* in cystic fibrosis. Curr. Opin. Pulm. Med..

[10] Epps QJ, Epps KL, Young DC, Zobell JT (2020). State of the art in cystic fibrosis pharmacology-Optimization of antimicrobials in the treatment of cystic fibrosis pulmonary exacerbations: I. Anti-methicillin-resistant *Staphylococcus aureus* (MRSA) antibiotics. Pediatr. Pulmonol..

[11] Otero LH (2013). How allosteric control of *Staphylococcus aureus* penicillin binding protein 2a enables methicillin resistance and physiological function. Proc. Natl Acad. Sci. USA.

[12] Casapao, A. M. et al. Clinical outcomes in patients with heterogeneous vancomycin-intermediate *Staphylococcus aureus* (hVISA) bloodstream infection. *Antimicrob. Agents Chemother.* **57**, 4252–4259 (2013). 10.1128/AAC.00380-13 PMC3754327 23796929

[13] Sotgiu G (2018). Efficacy and effectiveness of Ceftaroline Fosamil in patients with pneumonia: a systematic review and meta-analysis. Respir. Res..

[14] Jones RN, Mendes RE, Sader HS (2010). Ceftaroline activity against pathogens associated with complicated skin and skin structure infections: results from an international surveillance study. J. Antimicrob. Chemother..

[15] Sader, H. S., Rhomberg, P. R., Doyle, T. B., Flamm, R. K. & Mendes, R. E. Evaluation of the revised ceftaroline disk diffusion breakpoints when testing a challenge collection of methicillin-resistant *Staphylococcus aureus* isolates. *J. Clin. Microbiol.* **56**, e00777-18 (2018). 10.1128/JCM.00777-18 PMC6258841 30257898

[16] Clinical and Laboratory Standards Institute (CLSI). *M100 Performance Standards for Antimicrobial Susceptibility Testing* (Wayne, PA, 2018).

[17] Clinical and Laboratory Standards Institute/National Committee for Clinical Laboratory Standards (NCCLS). *M100-S25: Performance Standards for Antimicrobial Susceptibility Testing* (Villanova, PA, 2015).

[18] Cannavino CR (2016). Evolution of ceftaroline-resistant MRSA in a child with cystic fibrosis following repeated antibiotic exposure. Pediatr. Infect. Dis. J..

[19] Long SW (2014). PBP2a mutations causing high-level Ceftaroline resistance in clinical methicillin-resistant *Staphylococcus aureus* isolates. Antimicrob. Agents Chemother..

[20] Fernandez R, Paz LI, Rosato RR, Rosato AE (2014). Ceftaroline is active against heteroresistant methicillin-resistant *Staphylococcus aureus* clinical strains despite associated mutational mechanisms and intermediate levels of resistance. Antimicrob. Agents Chemother..

[21] Mendes RE (2012). Characterization of methicillin-resistant *Staphylococcus aureus* displaying increased MICs of ceftaroline. J. Antimicrob. Chemother..

[22] Alm, R. A. et al. Analysis of *Staphylococcus aureus* clinical isolates with reduced susceptibility to ceftaroline: an epidemiological and structural perspective. *J. Antimicrob. Chemother.* **69**, 2065–2075 (2014). 10.1093/jac/dku114 24777906

[23] Strommenger B, Layer F, Klare I, Werner G (2015). Pre-use susceptibility to ceftaroline in Clinical *Staphylococcus aureus* isolates from Germany: is there a non-susceptible pool to be selected?. PLoS ONE.

[24] Schaumburg F, Peters G, Alabi A, Becker K, Idelevich EA (2016). Missense mutations of PBP2a are associated with reduced susceptibility to ceftaroline and ceftobiprole in African MRSA. J. Antimicrob. Chemother..

[25] Kelley WL, Jousselin A, Barras C, Lelong E, Renzoni A (2015). Missense mutations in PBP2A Affecting ceftaroline susceptibility detected in epidemic hospital-acquired methicillin-resistant *Staphylococcus aureus* clonotypes ST228 and ST247 in Western Switzerland archived since 1998. Antimicrob. Agents Chemother..

[26] Jones RN, Flamm RK, Sader HS, Stilwell MG (2013). Interim susceptibility testing for ceftaroline, a new MRSA-active cephalosporin: selecting potent surrogate beta-lactam markers to predict ceftaroline activity against clinically indicated species. Diagn. Microbiol. Infect. Dis..

[27] Antonopoulos DA (2019). PATRIC as a unique resource for studying antimicrobial resistance. Brief. Bioinform.

[28] Pinho MG, Errington J (2005). Recruitment of penicillin-binding protein PBP2 to the division site of *Staphylococcus aureus* is dependent on its transpeptidation substrates. Mol. Microbiol..

[29] Pinho MG, Filipe SR, de Lencastre H, Tomasz A (2001). Complementation of the essential peptidoglycan transpeptidase function of penicillin-binding protein 2 (PBP2) by the drug resistance protein PBP2A in *Staphylococcus aureus*. J. Bacteriol..

[30] Reed P (2015). *Staphylococcus aureus* survives with a minimal peptidoglycan synthesis machine but sacrifices virulence and antibiotic resistance. PLoS. Pathog..

[31] Karinou, E., Schuster, C. F., Pazos, M., Vollmer, W. & Grundling, A. Inactivation of the monofunctional peptidoglycan glycosyltransferase SgtB allows *Staphylococcus aureus* to survive in the absence of lipoteichoic acid. *J. Bacteriol*. **201**, e00574-18 (2019). 10.1128/JB.00574-18 PMC6287468 30322854

[32] Komatsuzawa H (2000). Tn551-mediated insertional inactivation of the fmtB gene encoding a cell wall-associated protein abolishes methicillin resistance in *Staphylococcus aureus*. J. Antimicrob. Chemother..

[33] Garofalo A (2012). The length of the *Staphylococcus aureus* protein A polymorphic region regulates inflammation: impact on acute and chronic infection. J. Infect. Dis..

[34] Tan CM (2012). Restoring methicillin-resistant *Staphylococcus aureus* susceptibility to beta-lactam antibiotics. Sci. Transl. Med..

[35] Dumitrescu O (2011). Beta-lactams interfering with PBP1 induce Panton-Valentine leukocidin expression by triggering sarA and rot global regulators of *Staphylococcus aureus*. Antimicrob. Agents Chemother..

[36] Davies TA (2007). Binding of ceftobiprole and comparators to the penicillin-binding proteins of *Escherichia coli*, *Pseudomonas aeruginosa*, *Staphylococcus aureus*, and *Streptococcus pneumoniae*. Antimicrob. Agents Chemother..

[37] Pereira SF, Henriques AO, Pinho MG, de Lencastre H, Tomasz A (2007). Role of PBP1 in cell division of *Staphylococcus aureus*. J. Bacteriol..

[38] Pereira SF, Henriques AO, Pinho MG, de LH, Tomasz A (2009). Evidence for a dual role of PBP1 in the cell division and cell separation of *Staphylococcus aureus*. Mol. Microbiol..

[39] Rosato AE, Craig WA, Archer GL (2003). Quantitation of mecA transcription in oxacillin-resistant *Staphylococcus aureus* clinical isolates. J. Bacteriol..

[40] Rosato AE (2003). mecA-blaZ corepressors in clinical *Staphylococcus aureus* isolates. Antimicrob. Agents Chemother..

[41] Plata KB, Riosa S, Singh CR, Rosato RR, Rosato AE (2013). Targeting of PBP1 by beta-lactams determines recA/SOS response activation in heterogeneous MRSA clinical strains. PLoS ONE.

[42] Desbois AP, Coote PJ (2011). Wax moth larva (Galleria mellonella): an in vivo model for assessing the efficacy of antistaphylococcal agents. J. Antimicrob. Chemother..

[43] Esposito S (2019). Antimicrobial treatment of *Staphylococcus aureus* in patients with cystic fibrosis. Front Pharm..

[44] Mustafa MH (2016). Antimicrobial susceptibility of *Pseudomonas aeruginosa* isolated from cystic fibrosis patients in Northern Europe. Antimicrob. Agents Chemother..

[45] Chalhoub H (2016). High-level resistance to meropenem in clinical isolates of *Pseudomonas aeruginosa* in the absence of carbapenemases: role of active efflux and porin alterations. Int J. Antimicrob. Agents.

[46] Delfino E (2018). Pharmacokinetics of high-dose extended-infusion meropenem during pulmonary exacerbation in adult cystic fibrosis patients: a case series. N. Microbiol.

[47] Barsky EE (2018). Ceftaroline pharmacokinetics and pharmacodynamics in patients with cystic fibrosis. J. Cyst. Fibros..

[48] Suwantarat N (2018). Frequency of small-colony variants and antimicrobial susceptibility of methicillin-resistant *Staphylococcus aureus* in cystic fibrosis patients. Diagn. Microbiol. Infect. Dis..

[49] Autry EB (2016). Pharmacokinetic and pharmacodynamic analyses of ceftaroline in adults with cystic fibrosis. Pharmacotherapy.

[50] Singh R (2017). Ceftaroline efficacy against high-MIC clinical *Staphylococcus aureus* isolates in an in vitro hollow-fibre infection model. J. Antimicrob. Chemother..

[51] Stryjewski ME, Jones RN, Corey GR (2015). Ceftaroline: clinical and microbiology experience with focus on methicillin-resistant *Staphylococcus aureus* after regulatory approval in the USA. Diagn. Microbiol. Infect. Dis..

[52] Jacobs MR (2010). Activity of ceftaroline against recent emerging serotypes of *Streptococcus pneumoniae* in the United States. Antimicrob. Agents Chemother..

[53] Sader HS, Flamm RK, Streit JM, Carvalhaes CG, Mendes RE (2018). Antimicrobial activity of ceftaroline and comparator agents tested against organisms isolated from patients with community-acquired bacterial pneumonia in Europe, Asia, and Latin America. Int. J. Infect. Dis..

[54] Pfaller, M. A., Mendes, R. E., Duncan, L. R., Flamm, R. K. & Sader, H. S. In vitro activities of ceftaroline and comparators against *Streptococcus pneumoniae* isolates from U.S. hospitals: results from seven years of the AWARE surveillance program (2010 to 2016). *Antimicrob. Agents Chemother.* **62**, e01555-17 (2018). 10.1128/AAC.01555-17 PMC5786790 29158271

[55] Andrey DO (2017). Antimicrobial activity of ceftaroline against methicillin-resistant *Staphylococcus aureus* (MRSA) isolates collected in 2013–2014 at the Geneva University Hospitals. Eur. J. Clin. Microbiol Infect. Dis..

[56] Barbhaiya RH (1992). Pharmacokinetics of cefepime after single and multiple intravenous administrations in healthy subjects. Antimicrob. Agents Chemother..

[57] Butterfield JM (2014). Pharmacokinetics and pharmacodynamics of extended-infusion piperacillin/tazobactam in adult patients with cystic fibrosis-related acute pulmonary exacerbations. J. Antimicrob. Chemother..

[58] Nikolenko SI, Korobeynikov AI, Alekseyev MA (2013). BayesHammer: Bayesian clustering for error correction in single-cell sequencing. BMC Genomics.

[59] Bankevich A (2012). SPAdes: a new genome assembly algorithm and its applications to single-cell sequencing. J. Comput. Biol..

[60] Brettin T (2015). RASTtk: a modular and extensible implementation of the RAST algorithm for building custom annotation pipelines and annotating batches of genomes. Sci. Rep..

[61] Edgar RC (2004). MUSCLE: a multiple sequence alignment method with reduced time and space complexity. BMC Bioinforma..

[62] Edgar RC (2004). MUSCLE: multiple sequence alignment with high accuracy and high throughput. Nucleic Acids Res..

[63] Talavera G, Castresana J (2007). Improvement of phylogenies after removing divergent and ambiguously aligned blocks from protein sequence alignments. Syst. Biol..

[64] Cock PJ (2009). Biopython: freely available Python tools for computational molecular biology and bioinformatics. Bioinformatics.

[65] Stamatakis A (2014). RAxML version 8: a tool for phylogenetic analysis and post-analysis of large phylogenies. Bioinformatics.

