# Supplementary material for: Carbapenems drive the collateral resistance to ceftaroline in cystic fibrosis patients with MRSA
Source: Commun Biol. 2020 Oct 22;3:599. doi: 10.1038/s42003-020-01313-5 (PMC7582194; doi:10.1038/s42003-020-01313-5)
Supplement: Supplementary file 1 — Description of additional supplementary files [file 42003_2020_1313_MOESM1_ESM.docx]

Description of Additional Supplementary Files

Supplementary Data 1-3: Source data underlying plots shown in figures.
