# Supplementary material for: Carbapenems drive the collateral resistance to ceftaroline in cystic fibrosis patients with MRSA
Source: Commun Biol. 2020 Oct 22;3:599. doi: 10.1038/s42003-020-01313-5 (PMC7582194; doi:10.1038/s42003-020-01313-5)
Supplement: Supplementary file 5 — Reporting Summary [file 42003_2020_1313_MOESM5_ESM.pdf]

nature research

Corresponding author(s): Rosato
Last updated by author(s): 07-06-2020

Reporting Summary

Nature Research wishes to improve the reproducibility of the work that we publish. This form provides structure for consistency and transparency in reporting. For further information on Nature Research policies, see our Editorial Policies and the Editorial Policy Checklist.

Statistics

For all statistical analyses, confirm that the following items are present in the figure legend, table legend, main text, or Methods section.

n/a	Confirmed	
☐	☒	The exact sample size (*n*) for each experimental group/condition, given as a discrete number and unit of measurement
☐	☒	A statement on whether measurements were taken from distinct samples or whether the same sample was measured repeatedly
☐	☒	The statistical test(s) used AND whether they are one- or two-sided<br>*Only common tests should be described solely by name; describe more complex techniques in the Methods section.*
☐	☒	A description of all covariates tested
☐	☒	A description of any assumptions or corrections, such as tests of normality and adjustment for multiple comparisons
☐	☒	A full description of the statistical parameters including central tendency (e.g. means) or other basic estimates (e.g. regression coefficient) AND variation (e.g. standard deviation) or associated estimates of uncertainty (e.g. confidence intervals)
☐	☒	For null hypothesis testing, the test statistic (e.g. *F*, *t*, *r*) with confidence intervals, effect sizes, degrees of freedom and *P* value noted<br>*Give P values as exact values whenever suitable.*
☒	☐	For Bayesian analysis, information on the choice of priors and Markov chain Monte Carlo settings
☒	☐	For hierarchical and complex designs, identification of the appropriate level for tests and full reporting of outcomes
☒	☐	Estimates of effect sizes (e.g. Cohen's *d*, Pearson's *r*), indicating how they were calculated

Our web collection on statistics for biologists contains articles on many of the points above.

Software and code

Policy information about availability of computer code

Data collection	Data collection was not linked to any commercial software. S. aureus strains were isolated from sputum samples from cystic fibrosis patients, either WT or small colony variant phenotypes, and were obtained from three academic medical institutions with large CF populations: the Center of Global Infectious Diseases (Seattle, WA), UW Health (Madison, WI), and Houston Methodist Hospital (Houston, TX). Strains were selected from stored isolate banks by investigators at these institutions. These strains were identified from patient sputum at the time of clinical care, characterized in the microbiology laboratory, and stored at -80C until analysis. Only one strain per patient was included. Data collection was not linked to any commercial software.
Data analysis	Reads for 75 S. aureus sequenced genomes were assembled using The Pathosystems Resource Integration Center (PATRIC) genome assembly service using the “Full Spades” pipeline, which uses Bayes Hammerfor error correction and SPAdes for the assembly. All genomes were annotated using the PATRIC annotation service in May of 2019 and are available to the public.

For manuscripts utilizing custom algorithms or software that are central to the research but not yet described in published literature, software must be made available to editors and reviewers. We strongly encourage code deposition in a community repository (e.g. GitHub). See the Nature Research guidelines for submitting code & software for further information.

Data

Policy information about availability of data

All manuscripts must include a data availability statement. This statement should provide the following information, where applicable:

- Accession codes, unique identifiers, or web links for publicly available datasets
- A list of figures that have associated raw data
- A description of any restrictions on data availability

Data availability

The datasets generated during and/or analysed during the current study are available in the following repositories. All raw genome sequence data have been submitted to the The Pathosystems Resource Integration Center (PATRIC) database and is publicly available.
DNA sequencing data is available at Sequence Read Archive (SRA; https://www.ncbi.nlm.nih.gov/sra) under bioproject accession PRJNA635117. All the data produced for this study including in-vitro generated mutants are available.

The datasets generated during and/or analysed during the current study are available in the following repositories. All raw genome sequence data have been submitted to the The Pathosystems Resource Integration Center (PATRIC) database and is publicly available.
DNA sequencing data is available at Sequence Read Archive (SRA; https://www.ncbi.nlm.nih.gov/sra) under bioproject accession PRJNA635117. All the data produced for this study including in-vitro generated mutants are available.

Field-specific reporting

Please select the one below that is the best fit for your research. If you are not sure, read the appropriate sections before making your selection.

☒ Life sciences ☐ Behavioural & social sciences ☐ Ecological, evolutionary & environmental sciences

For a reference copy of the document with all sections, see nature.com/documents/nr-reporting-summary-flat.pdf

Life sciences study design

All studies must disclose on these points even when the disclosure is negative.

Sample size	We screened 335 samples to have a statistical significant observations between the strains provided by the three CF centers ans similar sample size around 100 strains per center.
Data exclusions	NA
Replication	All the attempts all data replication has been successful, both technical and biological replicates.
Randomization	Allocation of strains was random.
Blinding	All investigators were blinded .

Reporting for specific materials, systems and methods

We require information from authors about some types of materials, experimental systems and methods used in many studies. Here, indicate whether each material, system or method listed is relevant to your study. If you are not sure if a list item applies to your research, read the appropriate section before selecting a response.

Materials & experimental systems

n/a	Involved in the study	
☒	☐	Antibodies
☒	☐	Eukaryotic cell lines
☒	☐	Palaeontology and archaeology
☐	☒	Animals and other organisms
☒	☐	Human research participants
☒	☐	Clinical data
☒	☐	Dual use research of concern

Methods

n/a	Involved in the study	
☒	☐	ChIP-seq
☒	☐	Flow cytometry
☒	☐	MRI-based neuroimaging

Animals and other organisms

Policy information about studies involving animals; ARRIVE guidelines recommended for reporting animal research

Laboratory animals	Mice of the NOD.Cg-Prkdcscid Il2rgtm1Wjl/SzJ (NSG) strain were used in this study (The Jackson Laboratory, Bar Harbor, ME). Mice of the NOD.Cg-Prkdcscid Il2rgtm1Wjl/SzJ (NSG) strain were used in this study (The Jackson Laboratory, Bar Harbor, ME). Animals from both sex were fed with standard small laboratory animal diet, and no restrictions were applied.
Wild animals	NA
Field-collected samples	NA
Ethics oversight	All animal studies were approved by the Institutional Animal Care and Use Committee of the Houston Methodist Research Institute, protocol numbers AUP 0320-0023 and AUP 1018-0061. To ensure protection and proper manipulation of animals, experiments were

performed by trained personnel at the animal facility of the Houston Methodist Research Institute.

Note that full information on the approval of the study protocol must also be provided in the manuscript.
